# Do No Harm: Moving Beyond Weight Loss to Emphasize Physical Activity at Every Size

**DOI:** 10.5888/pcd14.170006

**Published:** 2017-04-20

**Authors:** Emily Dollar, Margit Berman, Anna M. Adachi-Mejia

**Affiliations:** 1 Geisel School of Medicine at Dartmouth, Lebanon, New Hampshire; 2 Minnesota School of Professional Psychology at Argosy University, Eagan, Minnesota; 3 Health Promotion Research Center at Dartmouth (HPRCD), Lebanon, New Hampshire; 4 The Dartmouth Institute for Health Policy and Clinical Practice, Lebanon, New Hampshire; 5 Cancer Control Research Program, Norris Cotton Cancer Center, Lebanon, New Hampshire

People with a high body mass index (BMI) encounter stigma in the health care setting, which can negatively affect health outcomes. We suggest that providers can reduce this stigma and improve outcomes by moving away from a focus on weighing patients and counseling about weight loss and toward emphasizing healthy behaviors. We propose that a conversation between health care provider and patient about increasing the patient’s physical activity rather than focusing on body weight will enable providers to promote healthful behaviors, decrease stigma, and strengthen patient–provider trust and rapport.

## Shifting the Conversation From Body Weight to Behavior

Health care providers are trained to treat a patient’s weight as though it were a vital sign. Weighing patients and calculating body mass index (BMI) are standard practices at most clinical visits, as routine as checking blood pressure. Medicare Meaningful Use and other guidelines often require health care providers to collect these data. However, the process can be stigmatizing for patients of any size. The stigma associated with a health care provider’s assessment of body weight is associated with medication nonadherence, mistrust of the provider, and avoidance of medical care (1). Although a weight or BMI measurement is sometimes necessary for medical reasons, discussions about these numbers are potentially harmful (2). We suggest that providers engage in conversations that emphasize health behaviors rather than weight, such as increasing or maintaining physical activity. Patient-centered counseling that emphasizes behavior over body size can minimize the risk of stigma and help patients build health goals in a collaborative, supportive environment.

## What Have We Gained With Weight Loss Counseling?

Health care providers, especially in primary care, increasingly feel a responsibility to address the obesity epidemic and help their patients achieve a healthy weight. However, we have yet to see evidence of how providers can consistently, effectively, and reliably affect their patient’s body size over the long term. Sustained weight loss of greater than 5% of body weight is rare. Even when people adhere to strict, high-volume exercise, weight loss varies (3). Both in naturalistic, longitudinal samples and in randomized controlled trials, various weight-loss efforts and strategies lead to long-term weight gain and the onset of obesity (4,5). Furthermore, a person’s perception (or in children, their parents’ perception) that they are overweight also leads to long-term weight gain, not weight maintenance or loss, regardless of their BMI (2), suggesting that awareness-raising conversations about body weight can do more harm than good.

Providers hoping to address the obesity epidemic have few evidence-based tools or resources to draw on to effectively counsel their patients and are often left struggling with how to have this conversation about body weight in the best way. No matter what the intention, if conversations encouraging weight loss are perceived as stigmatizing or harmful, they will lead to failure, both for the patient–provider relationship and for the patient’s health improvement efforts.

## Moving Toward Meaningful Health Promotion

Programs such as the Health at Every Size movement promote health and wellness at all weights (6). Adopting this approach has the potential both to improve patient outcomes and decrease the patient’s perceived stigmatization in the eyes of the provider, enhancing the patient–provider relationship. To be successful, the paradigm of health at any weight needs to be incorporated into all levels of medical education and training. Inclusive, nonstigmatizing approaches to health promotion must also acknowledge the social and economic determinants of health and take into consideration the patient’s lived environment. Only by taking such an approach can health care providers help patients achieve meaningful and sustainable health goals.

An important component of meaningful health promotion should be a conversation about increasing and maintaining physical activity and reducing sedentary behavior. Increasingly, studies support the concept that cardiorespiratory fitness, regardless of body weight, is the key indicator for reducing mortality risk (7). Substantial evidence shows that regular physical activity can reduce the risk of chronic diseases, including diabetes, coronary heart disease, and Alzheimer’s disease (8). The American College of Sports Medicine’s “Exercise is Medicine” initiative advocates making physical activity a vital sign that should be assessed at every visit (9). Focusing on the physical activity vital sign rather than body weight can empower providers to structure a conversation about health concerns in a way that is productive and proactive and allows for patient-initiated goal setting. A conversation about increasing and maintaining physical activity and reducing sedentary behavior also provides an opportunity for providers to talk with patients about their lived environment and the barriers that prevent them from adopting healthy behaviors. Emphasizing the physical activity vital sign over BMI assessment shifts patient assessment toward more meaningful, behavior-based health indicators and avoids the risk of judging patients on the basis of personal habits alone. 

Let’s welcome patients who have avoided and feared going to the doctor because of feeling shame about their size. Let’s acknowledge the social determinants of health that affect a person’s body size. Let’s learn how to communicate with patients about these determinants and empower them to make healthy choices within the context of their own lives. Rather than harming patients with stigmatizing measurements that limit our ability to have a productive relationship, let’s focus our precious clinical time on helping patients to engage in active lifestyles. The result may be better outcomes in patient health and patient trust and improved patient–provider relationships. 
